# Supplementary material for: Sewage Pollution Promotes the Invasion-Related Traits of Impatiens glandulifera in an Oligotrophic Habitat of the Sharr Mountain (Western Balkans)
Source: Plants (Basel). 2021 Dec 20;10(12):2814. doi: 10.3390/plants10122814 (PMC8708665; doi:10.3390/plants10122814)
Supplement: Supplementary file 1 [file plants-10-02814-s001.zip › plants-1500447-supplementary.pdf]

Sewage pollution promotes the invasion-related traits of *Impatiens glandulifera* in an oligotrophic habitat of the Sharr mountain (Western Balkans)

Table S1: List of species with the estimated cover abundance > 1% spontaneously occurring in the resident vegetation invaded by *Impatiens glandulifera* (Štrpce/Shtërpcë municipality, mountainous area in the Southeast Kosovo). Sites/habitats are coded as in Table 1. Nomenclature follows the W3 TROPICOS database of the Missouri Botanical Garden.

Species in resident vegetation	Invaded habitats		
	A	B	C
Agrimonia eupatoria L.		+	
Agrostis alba L.	+	+	
Ajuga reptans L.			+
Alnus glutinosa (L.) Geartn.	+	+	
Amaranhus retroflexus L.	+		+
Anchusa officinalis (L.) Gouan			+
Anthriscus sylvestris (L.) Hoffm.	+	+	
Arctium lappa L.	+		+
Aruncus dioicus (Walter) Fernald		+	
Astrantia minor L.		+	
Barbarea vulgaris W.T. Alton	+	+	+
Bidens tripartita L.	+	+	
Bromus sterilis L.			+
Calystegia sepium (L.) R. Br.	+	+	
Cardamine bulbifera (L.) Crantz			
Carex sylvatica Huds.	+	+	
Carum carvi L.		+	
Cephalanthera rubra (L.) Rich.		+	
Chaerophyllum aureum L.	+	+	
Chamaenerion angustifolium (L.) Scop.		+	
Chelidonium majus L.	+	+	
Chenopodium album L.	+		+
Cichorium intybus L.			+
Cirsium arvense (L.) Scop.	+		+
Clematis vitalba L.	+	+	
Cornus sanguinea L.	+	+	
Crepis biennis L.	+	+	+
Cruciata laevipes Opiz		+	+
Dactylis glomerata L.	+		+
Daucus carota L.			+
Elymus repens (L.) Gould			+
Erigeron canadensis L.			+
Euphorbia helioscopia L.		+	+
Fallopia convolvulus (L.) Á. Löve			+
Filipendula ulmaria (L.) Maxim.		+	
Fraxinus ornus L.	+	+	

Galium aparine L.		+	+
Geranium mole L.			+
Geranium phaeum L.		+	
Geum urbanum L.		+	+
Glechoma hederacea L.		+	
Heracleum sphondylium L.		+	
Hordeum murinum L.			+
Impatiens komarovii Pobed.		+	
Lamium maculatum L.	+	+	+
Lythrum salicaria L.		+	
Malva sylvestris L.			+
Medicago arabica (L.) Huds.			+
Melilotus officinalis (L.) Lam.			+
Melittis melissophyllum L.		+	
Mentha longifolia (L.) Huds.	+	+	+
Myosotis sparsiflora J.C. Mikan		+	
Myosotis sylvatica Ehrh. ex Hoffm.	+	+	
Persicaria lapathifolia (L.) Delarbre	+		+
Petasites hybridus (L.) G. Gaertn., B. May & Scherb.	+	+	
Plantago lanceolata L.			+
Polygonum aviculare L.			+
Populus nigra L.	+	+	
Potentilla reptans L.			+
Prunus cerasifera Ehrh.	+		
Ranunculus repens L.	+	+	
Ranunculus sceleratus L.	+		
Rorippa sylvestris (L.) Besser			
Rosa canina L.		+	+
Rubus caesius L.	+	+	+
Rumex crispus L.	+		+
Salix caprea L.	+	+	
Salix elaeagnos Scop.	+	+	
Salix viminalis L.		+	
Salvia verticillata L.			+
Sambucus ebulus L.	+	+	+
Sambucus nigra L.	+	+	
Saponaria officinalis L.	+	+	+
Scutellaria columnae All.		+	
Setaria italica(L.) P. Beauv.			+
Sherardia arvensis L.		+	
Silene latifolia Poir.	+		+
Sisymbrium officinale (L.) Scop.			+
Sonchus arvensis L.	+		+
Stellaria holostea L.		+	
Stenactis annua (L.) Cass. ex Less.			+
Telekia speciosa (Schreb.) Baumg.	+	+	
Thalictrum aquilegiifolium L.		+	

Trifolium pratense L.			+
Urtica dioica L.	+	+	+
Verbascum phlomoides L.			+
Veronica chamaedrys L.	+	+	+
Vicia cracca L.		+	+

Table S2: Selected soil parameters of non-invaded soils of (semi)natural meadows outside of the flooding zone of the Lepenac river.

Soil parameter	Median	Range
pH	6.9	6.6 – 7.3
Available P (mg kg^{-1})	10.3	7.3 – 18.2
Available K (mg kg^{-1})	196.1	123.5 – 250.3
Total N (%)	0.38	0.24 – 0.51

Meadows were chiefly dominated by *Danthonia alpina, Koeleria pyramidata, Dactylis glomerata* or *Arrhenatherum elatius*. Results of 15 samples are presented.

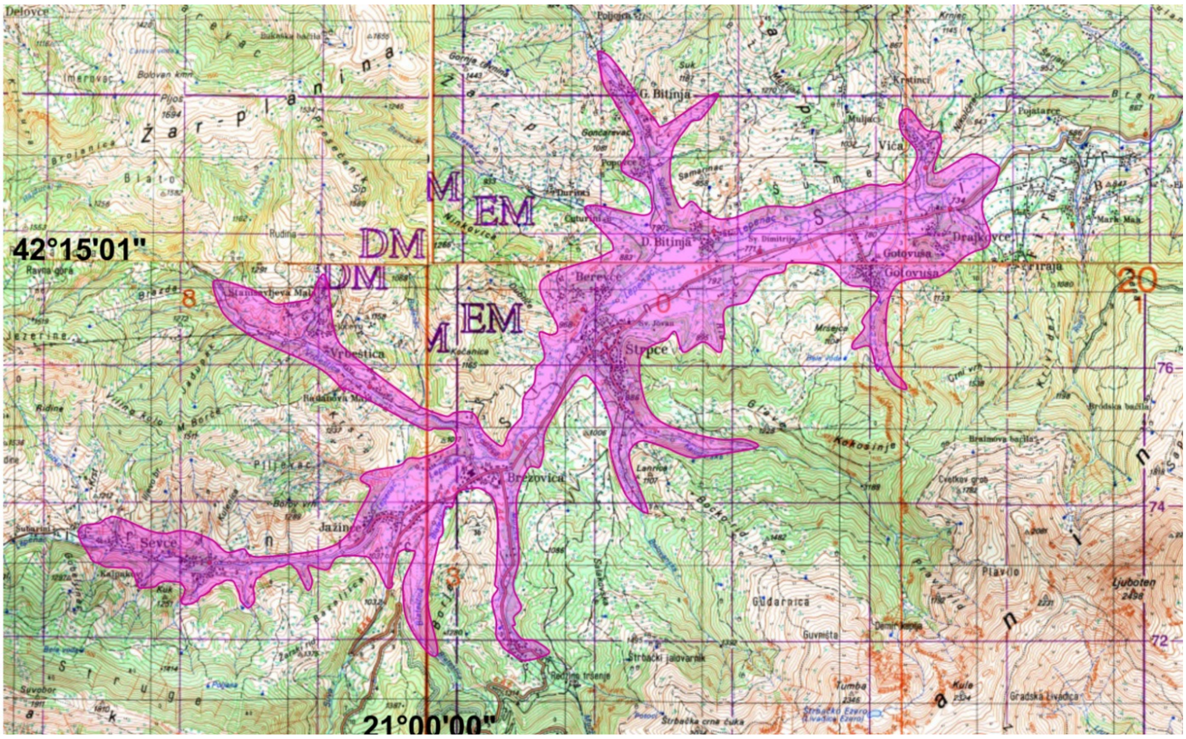

Figure S1: Distribution of the well-established *Impatiens glandulifera* stands (purple shade) in the source area of the Lepenac river, in a valley of the Sharr mountain range, southeast Kosovo. The delineated area encompasses individual observations of “significant” (>10 m^2, or more than 50 individuals) stands of Himalayan balsam separated by <100 m physical distance. Basal topographic map 1:50000 (Vojnogeografski Institut, Belgrade, 1985).
